# Myoblast transplantation improves cardiac function after myocardial infarction through attenuating inflammatory responses

**DOI:** 10.18632/oncotarget.18244

**Published:** 2017-05-27

**Authors:** Bo Wang, Likui Zhang, Hao Cao, Junqi Yang, Manya Wu, Yali Ma, Huimin Fan, Zhenzhen Zhan, Zhongmin Liu

**Affiliations:** ^1^ Key Laboratory of Arrhythmias of the Ministry of Education of China and Institute of Heart Failure, Shanghai East Hospital, Tongji University School of Medicine, Shanghai 200120, China; ^2^ Department of Cardiac Surgery and Institute of Heart Failure, Shanghai East Hospital, Tongji University School of Medicine, Shanghai 200120, China

**Keywords:** myoblasts, transplantation, myocardial infarction, cardiac function, inflammation

## Abstract

Myocardial infarction (MI) is a highly prevalent cardiac emergency, which results in adverse cardiac remodeling and then exacerbates progressive heart failure. Inflammatory responses in cardiac tissue after MI is necessary for myocardium repair and wound healing. However, the excessive inflammation is also a key component of subsequent heart failure pathology. Myoblast transplantation after MI have been fulfilled attractive effects on cardiac repair, but the complications of transplantation and the underlying mechanisms have not been fully elucidated. Here, we found that human myoblast transplantation into minipig myocardium decreased the infiltration of inflammatory cells, the expression levels of many pro-inflammatory genes and the activation of inflammation-related signal pathways, while upregulated the expression levels of anti-inflammatory genes such as IL-10 in cardiac tissue of minipig post-MI, which was contributed to the improved cardiac function, the decreased infarct area and the attenuated myocardial fibrosis. Moreover, co-culture of human myoblasts inhibited the production of IL-1β and TNF-α as well as activation of MAPK and NF-κB signaling pathway induced by damage-associated molecular patterns such as HMGB1 and HSP60 in human THP-1 cells, which was partially attributed to the up-regulated production of IL-10. Collectively, these results indicate that myoblast transplantation ameliorates heart injury and improves cardiac function post-MI through inhibiting the inflammatory response, which provides the novel mechanism for myoblast transplantation therapy of MI.

## INTRODUCTION

Myocardial infarction (MI) mainly caused by coronary artery occlusion has higher morbidity and mortality, and pathological progress of which includes ischemia-reperfusion injury, wound healing responses and cardiac remodeling [1]. Reperfusion injury leads to necrosis of cardiac myocytes and release of endogenous damage-associated molecular patterns (DAMPs) which recruit inflammatory cells to the infarct area [1]. These inflammatory cells, such as neutrophils, monocytes and macrophages, can clear necrotic debris and also contribute to acute reperfusion injury by releasing excessive inflammatory cytokines, matrix metalloproteinases and reactive oxygen species, leading to the expansion of infarct area [2, 3]. In addition, inflammatory cells play a crucial role in the chronic phase, months and even years after the ischemic event, when low-grade inflammation persists in heart and peripheral organs [4]. Therefore, tight control and timely repression of this inflammatory response has the potential to limit reperfusion injury and improve outcome in acute MI event and chronic ischemic heart failure [5].

Over the past decades, various therapeutic methods including coronary artery bypass grafting (CABG), percutaneous coronary intervention (PCI) and cell-based transplantation rapidly blossom in ischemic heart diseases. Although CABG and PCI can restore blood flow, they are proved defective in risk of anesthesia, operation attack and have the possibility of augmenting inflammation [6–8]. Transplantation of adult bone marrow-derived stem and progenitor cells into the infarct myocardium improves infarct healing and the recovery of cardiac function after MI in experimental studies and in patients [9]. The transplanted cells may have effects on anti-apoptosis, pro-angiogenesis and anti-inflammation in a paracrine fashion [9, 10]. However, transplantation of stem and progenitor cells is limited because of their oncogenicity and fastidious culture condition [11].

Skeletal myoblasts (SkMs) are derived from satellite cells which locate between the sarcolemma and the basal lamina of myofibers. When muscle goes through overstretching, straining, trauma, myoblasts are activated to enter the cell cycle, proliferate and terminally differentiate into myofibers [12]. Based on these characteristics, myoblasts have been applied to cure muscular dystrophy and get desirable outcomes [13–15]. Compared with stem and progenitor cells, myoblasts embrace the advantages of facility of procurement, rapidity of expansion *in vitro*, resistance to ischemic conditions, no ethic restriction and low oncogenicity [12]. Thus, myoblast therapy for ischemia cardiovascular diseases is desired and carried out well on animal models in recent years [16–19]. Menasche et al. performed the first human transplantation of myoblasts in patients with heart failure [20, 21], and additional clinical trials also have reported that myoblasts injected into infarct myocardium of ischemic cardiomyopathy attenuated left ventricular remodeling and resulted in a amelioration in left ventricular dysfunction [22, 23].

Although myoblast transplantation has affirmative therapeutic effects, the underlying mechanisms remain not fully clear. In this study, we found that the expression levels of pro-inflammatory cytokines and the activation of inflammation-related signal pathway in heart tissues were markedly decreased in minipigs with MI and then subjected to myoblast transplantation. In addition, co-culture of human THP-1 cells with myoblasts resulted in the decreased production of inflammatory cytokines and increased IL-10 production of in THP-1 cells stimulated with recombinational HMGB1. Our results demonstrate that myoblast transplantation improves cardiac function through alleviating inflammatory responses post-MI.

## RESULTS

### Grafted myoblasts survive and proliferate in heart tissues of minipigs after MI

**Figure 1:**
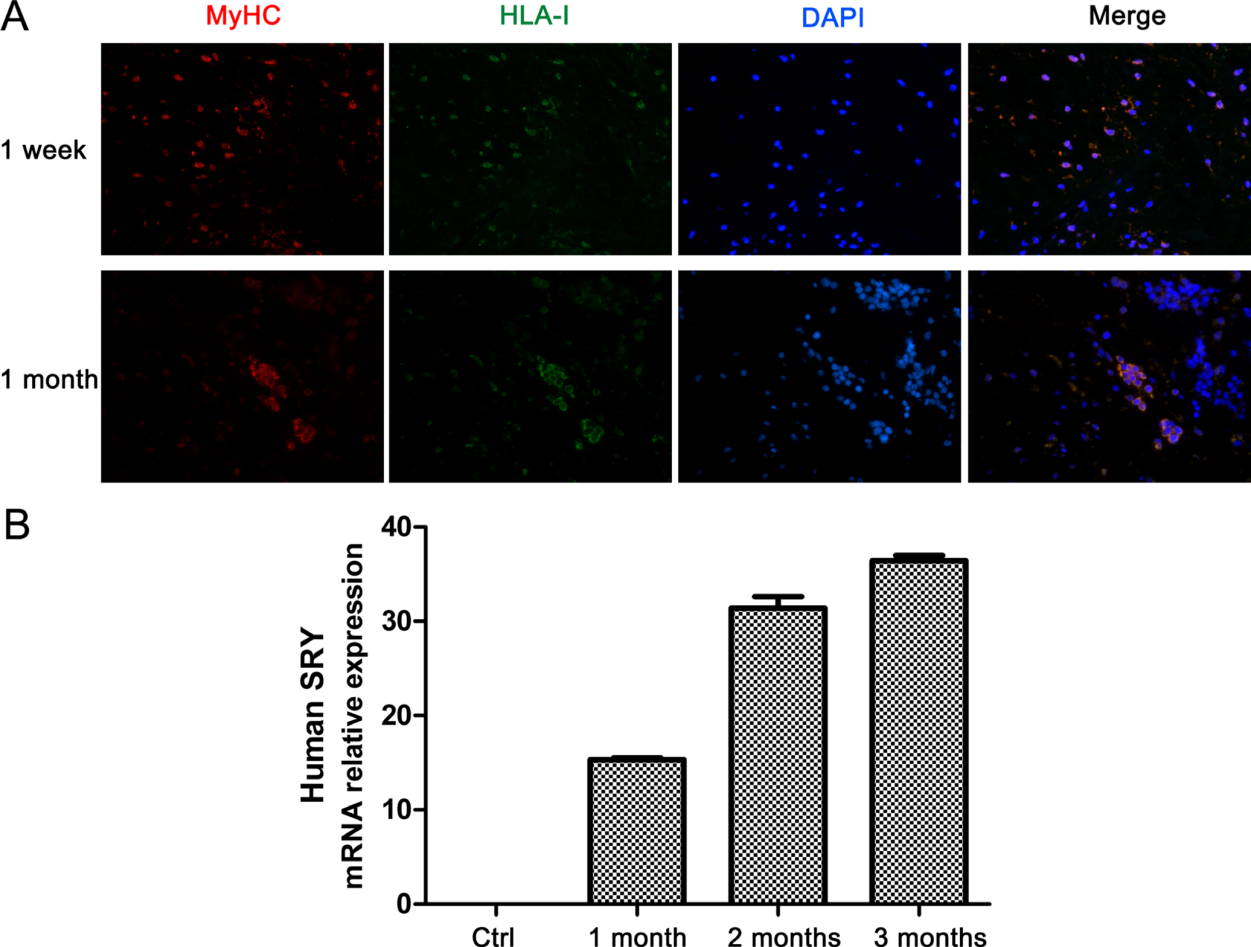
Grafted myoblasts survive and proliferate in heart tissue of minipig post-MI (**A**) Immunofluorescence staining of MyHC (red) and HLA-I (green) in heart sections from minipigs with MI at 1 week and 1 month after myoblast transplantation. Original magnification, ×400. (**B**) Q-PCR analysis of the expression of human Y chromosome in heart tissues of minipigs with MI followed by myoblast transplantation. Values are showed as the relative fold-change compared with control treatment groups (*n* = 6). Data are representative (A), or mean ± SEM (B) of 3 individual experiments.

Male human myoblasts were injected into border region of infarct zone in heart of minipigs with MI model constructed through ligation of left circumflex (LCx) coronary artery. First, antibodies to human myosin heavy chain (MyHC) and histocompatibility antigen class I (HLA-I) were used to stain grafted myoblasts in minipig heart undergoing transplantation. As shown in Figure 1A, double positive myoblasts which expressed MyHC and HLA-I indeed survived in heart tissue of minipig one week and one month after transplantation, and the double positive myoblasts had a clumped distribution in heart tissue one month after transplantation. Although the clumped myoblasts cannot be determined to be derived from one clone or just migrate to get together, it is sure that the grafted human myoblasts successfully survive and proliferate in minipig heart one month or even longer after transplantation. These results were further confirmed by detecting the mRNA expression of human Y chromosome which was significantly increased in minipig heart tissue along with the time post transplantation (Figure 1B).

### Myoblast transplantation ameliorates cardiac dysfunction post-MI

**Figure 2:**
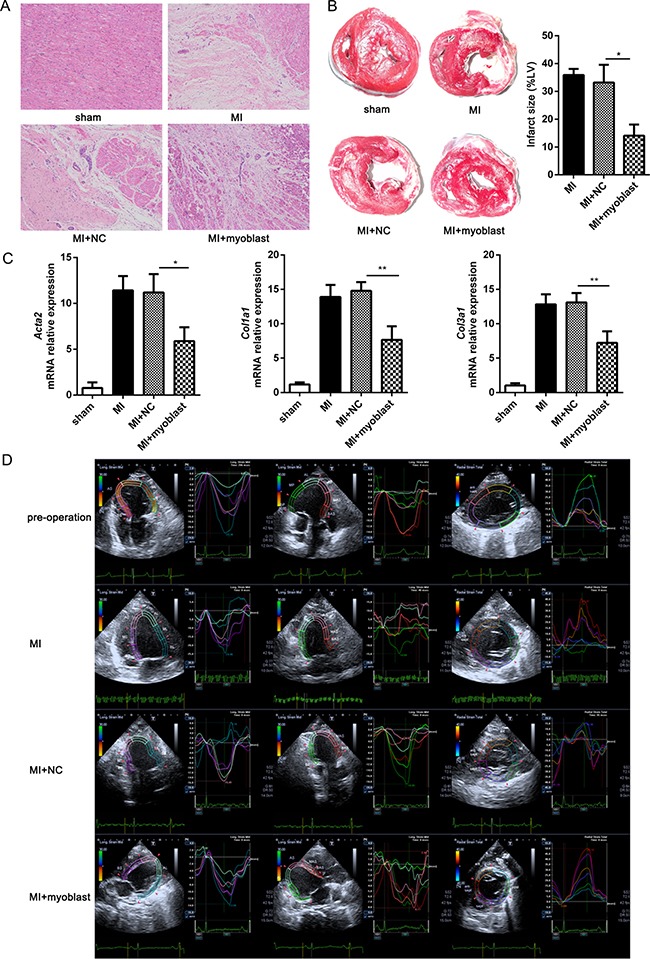
Myoblast transplantation improves cardiac function post-MI (**A**) Representative HE staining images of heart sections from minipigs with MI or sham operation at 1 month after myoblast transplantation, control treatment or left untreated. (**B**) Representative TTC staining images of heart left ventricular sections from minipigs treated as in (A), and infarct size was measured and showed as a percentage of total area of left ventricular myocardium. (**C**) Q-PCR analysis of the expression of *Acta2*, *Col1a1* and *Col3a1* in heart tissues from minipigs treated as in (A). (**D**) The measurement of longitudinal (left and middle panels) and radial (right panel) strain by 2D-STE of cardiac function of minipigs treated as in (A). n=6. Data are representative (A, B left, D), or mean ± SEM (B right, C) of 3 individual experiments. *P < 0.05, **P < 0.01.

Next, we observed the effect of grafted myoblasts on myocardial injury and fibrosis as well as cardiac function. One month after myoblast transplantation, hematoxylin & eosin (HE) staining of heart tissue showed that cardiomyocytes located around the peri-infarct region in heart with myoblast transplantation, while there were only myofibroblasts and collagen deposition around the peri-infarct region in control treatment (NC) group without myoblast transplantation (Figure 2A). Infarct area size was also significantly decreased in heart tissue of minipig with myoblast transplantation post-MI (Figure 2B). Consistently, the mRNA expression levels of fibrosis-related genes including *Acta2*, *Col1a1* and *Col3a1* in heart tissue were also markedly down-regulated in myoblast transplantation group compared with control treatment group (Figure 2C). Furthermore, left ventricular ejection fraction (EF) and fractional shortening (FS), the two main parameters which can effectively reflect the change of cardiac function, were markedly increased in myoblast transplantation group compared with control treatment group. However, left ventricular end-systolic internal diameter (LVIDs) and left ventricular end-systolic volume (LVESV) were significantly decreased in myoblast transplantation group. Left ventricular end-systolic posterior wall thickness (LVPWs) and left ventricular end-diastolic posterior wall thickness (LVPWd) were up-regulated in myoblast transplantation group compared with control treatment group and MI group (Figure 2D and Table 1). These data indicate that myoblast transplantation can attenuate myocardial injury and fibrosis, increase wall thickening at the ischemic area of heart, which in turn augments left ventricular systolic function and substantially improves cardiac function post-MI.

**Table 1:** Cardiac function assessment by 2-D echocardiography

2-D Echo	0 week	4 weeks		
	Sham (*n* = 6)	MI (*n* = 6)	NC (*n* = 6)	Myoblasts (*n* = 6)
LVIDd (mm)	32.3 ± 3.6	37.2 ± 4.2	37.4 ± 5.1	36.3 ± 3.8
LVIDs (mm)	21.3 ± 2.5	28.6 ± 3.2	27.1 ± 2.3	26.9 ± 2.9*
LVEDV (ml)	43.6 ± 4.7	56.8 ± 4.3	57.7 ± 4.9	55.2 ± 4.6
LVESV (ml)	16.2 ± 1.7	31.2 ± 2.4	31.3 ± 1.8	26.3 ± 2.1*
FS (%)	34.8 ± 3.6	21.4 ± 1.5	22.7 ± 1.2	27.6 ± 2.2*
EF (%)	64.1 ± 5.5	44.8 ± 4.2	46.3 ± 5.2	52.1 ± 4.7*
LVPWs (mm)	13.5 ± 0.3	6.5 ± 0.4	6.4 ± 0.5	10.4 ± 0.4*
LVPWd (mm)	8.3 ± 0.4	5.1 ± 0.3	5.3 ± 0.4	6.6 ± 0.2*

The assessment of cardiac function of minipigs with MI or sham operation at 1 month after myoblast transplantation, control treatment (NC) or left untreated. *P < 0.05 vs. control treatment group. LVIDd: left ventricular end-diastolic internal diameter; LVIDs: left ventricular end-systolic internal diameter; LVEDV: left ventricular end-diastolic volume; LVESV: left ventricular end-systolic volume; FS: fractional shortening; EF: ejection fraction; LVPWs: Left ventricular end-systolic posterior wall thickness; LVPWd: Left ventricular end-diastolic posterior wall thickness.

### The differentially expressed inflammation-related genes in heart tissue with myoblast transplantation

**Figure 3:**
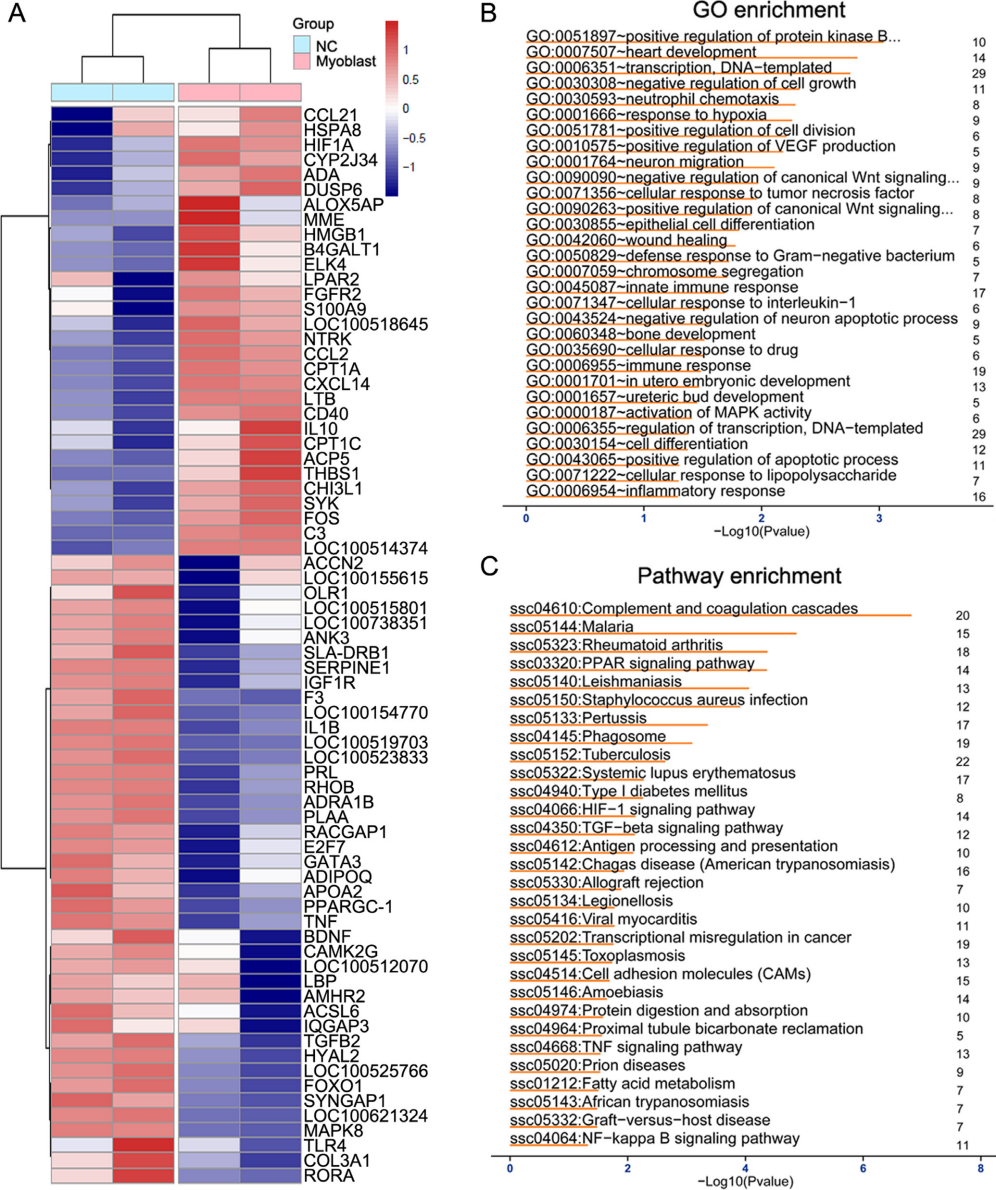
The expression profiles of inflammation-relative genes in heart tissues after myoblast transplantation (**A**) Heatmap showing 72 differentially expressed inflammation-related genes in heart tissues from minipigs with MI followed by myoblast transplantation or control treatment (NC). Red means an increase in expression level, whereas blue represents a decrease in expression level in each group. (**B**) GO enrichment analysis of differentially expressed genes. (**C**) KEGG enrichment analysis of differentially expressed genes.

To explore the mechanism by which myoblast transplantation promoted cardiac function recover after MI, we performed microarray analysis to observe differentially expressed gene profiles in peri-ischemia tissue from myoblast transplantation group and control treatment group. Hierarchical cluster of all differentially expressed genes (DEGs) and inflammation-related genes between these two groups were shown in heat map (Figure 3A and Supplementary Figure 1). There were 1041 up-regulated genes and 966 down-regulated genes (fold change ≥ 2) in peri-ischemia heart tissue of myoblast transplantation group compared with those in control treatment group (Supplementary Figure 1). Moreover, 45 of a set of 82 differentially expressed inflammation-related genes were significantly down-regulated, including pro-inflammatory cytokines such as TNF-α and IL-1β in heart tissue of minipig with myoblast transplantation (Figure 3A and Table 2). Some important genes involved in inflammation-related signal pathway, such as TLR4 and MAPK8, were also down-regulated in heart tissue of myoblast transplantation group, while anti-inflammatory gene IL-10 was up-regulated in heart tissue of myoblast transplantation group (Figure 3A and Table 2). We then arranged DEGs enrichment analysis in the Gene Ontology (GO) categories and Kyoto Encyclopedia of Genes and Genomes (KEGG) pathway. GO and pathway analysis showed top 30 significant pathways involving DEGs, some of which were closely consistent with down-regulated genes mentioned above (Figure 3B and 3C). These results reveal that myoblast transplantation can suppress the expression of pro-inflammatory gene in heart tissue post-MI.

**Table 2:** The differentially expressed inflammation-relative genes in heart tissue of minipigs treated with or without myoblast transplantation post-MI

Gene_Symbol	Gene_Title	Fold change (Myo/NC)
LOC100525766	serine/threonine-protein kinase PAK 1-like	0.018209086
ADIPOQ	adiponectin, C1Q and collagen domain containing	0.039351824
TLR4	Toll-like receptor 4	0.053853831
LOC100515801	Uncharacterized protein KIAA1383 homolog	0.07255373
IL1B	Interleukin 1 beta	0.154663889
LOC100519703	proteinase-activated receptor 2-like	0.185482438
TNF	Tumor necrosis factor	0.189656668
LBP	lipopolysaccharide binding protein	0.22179613
LOC100512070	glucose-6-phosphatase 3-like	0.251654884
LOC100154770	radiation-inducible immediate-early gene IEX-1-like	0.287977899
LOC100525766	serine/threonine-protein kinase PAK 1-like	0.293721545
ACSL6	acyl-CoA synthetase long-chain family member 6	0.29759855
PRL	prolactin	0.307770743
GATA3	GATA binding protein 3	0.311184563
ADRA1B	adrenoceptor alpha 1B	0.339089567
RHOB	ras homolog family member B	0.339561077
PPARGC-1	peroxisome proliferator activated receptor gamma, coactivator 1 alpha	0.345113903
IGF1R	insulin-like growth factor 1 receptor	0.364555886
AMHR2	anti-Mullerian hormone receptor, type II	0.369864566
COL3A1	Collagen, type III, alpha 1	0.370578657
LOC100155615	vasopressin-induced protein, 32kDa	0.377345245
CAMK2G	calcium/calmodulin-dependent protein kinase II gamma	0.381822705
SLA-DRB1	MHC class II histocompatibility antigen SLA-DRB1	0.387633403
ACCN2	acid-sensing (proton-gated) ion channel 1	0.391439296
MAPK8	Mitogen-activated protein kinase 8, JNK1	0.396123721
LBP	lipopolysaccharide binding protein	0.400341256
E2F7	E2F transcription factor 7	0.401168991
LOC100621324	heat shock-related 70 kDa protein 2-like	0.411213633
OLR1	oxidized low density lipoprotein (lectin-like) receptor 1	0.416144478
LOC100523833	fibroblast growth factor 13-like	0.419831988
BDNF	brain-derived neurotrophic factor	0.42688193
F3	coagulation factor III (thromboplastin, tissue factor)	0.439591227
PLAA	phospholipase A2-activating protein	0.446603047
TGFB2	transforming growth factor, beta 2	0.461156871
RORA	RAR-related orphan receptor A	0.46211275
FOXO1	forkhead box O1	0.462757924
SERPINE1	serpin peptidase inhibitor, clade E (nexin, plasminogen activator inhibitor type 1), member 1	0.463425071
RACGAP1	Rac GTPase activating protein 1	0.47786811
GATA3	GATA binding protein 3	0.487248416
IQGAP3	IQ motif containing GTPase activating protein 3	0.488153338
APOA2	apolipoprotein A-II	0.49103491
ANK3	ankyrin 3, node of Ranvier (ankyrin G)	0.49244312
SYNGAP1	synaptic Ras GTPase activating protein 1	0.496903153
HYAL2	hyaluronoglucosaminidase 2	0.498768065
LOC100738351	microtubule-associated protein tau-like	0.499859657
HIF1A	hypoxia inducible factor 1, alpha subunit (basic helix-loop-helix transcription factor)	2.006498392
LOC100515801	uncharacterized protein KIAA1383 homolog	2.027618033
HMGB1	high mobility group box 1	2.07995541
FOS	FBJ murine osteosarcoma viral oncogene homolog	2.108144854
CYP2J34	cytochrome P450, family 2, subfamily J, polypeptide 34	2.139775885
DUSP6	dual specificity phosphatase 6	2.205287882
LTB	lymphotoxin beta (TNF superfamily, member 3)	2.259628027
B4GALT1	UDP-Gal:betaGlcNAc beta 1,4- galactosyltransferase, polypeptide 1	2.280422277
THBS1	Thrombospondin 1	2.325687086
THBS1	thrombospondin 1	2.356147732
HSPA8	heat shock 70kDa protein 8	2.36804402
DUSP6	dual specificity phosphatase 6	2.421761848
SYK	spleen tyrosine kinase	2.422838934
CD40	CD40 molecule, TNF receptor superfamily member 5	2.478801936
CHI3L1	chitinase 3-like 1 (cartilage glycoprotein-39)	2.502965317
LOC100514374	dual specificity protein phosphatase 4-like	2.606314171
CPT1A	carnitine palmitoyltransferase 1A (liver)	2.609206269
ELK4	ETS-domain protein (SRF accessory protein 1)	2.812234501
C3	complement component 3	2.877851202
ADA	adenosine deaminase	2.901005438
LOC100519703	Proteinase-activated receptor 2-like	3.230116054
ACP5	acid phosphatase 5, tartrate resistant	3.33994883
IL10	interleukin 10	3.394530387
LOC100518643	Interleukin-33-like	3.693850557
ALOX5AP	arachidonate 5-lipoxygenase-activating protein	3.733013174
CPT1C	carnitine palmitoyltransferase 1C	3.738067187
THBS1	thrombospondin 1	4.004670655
FGFR2	fibroblast growth factor receptor 2	4.566747424
MME	membrane metallo-endopeptidase	4.625607971
CXCL14	chemokine (C-X-C motif) ligand 14	5.54041483
CCL21	chemokine (C-C motif) ligand 21	5.607199497
CCL2	chemokine (C-C motif) ligand 2	5.630495932
LPAR2	lysophosphatidic acid receptor 2	6.141604456
CXCL14	chemokine (C-X-C motif) ligand 14	7.729290554
S100A9	S100 calcium binding protein A9	8.279375777
NTRK3	Neurotrophic tyrosine kinase, receptor, type 3	10.19038462
THBS1	thrombospondin 1	14.95862849

Myo: myoblasts; NC, control treatment group.

### Myoblasts attenuate inflammatory responses post-MI

**Figure 4:**
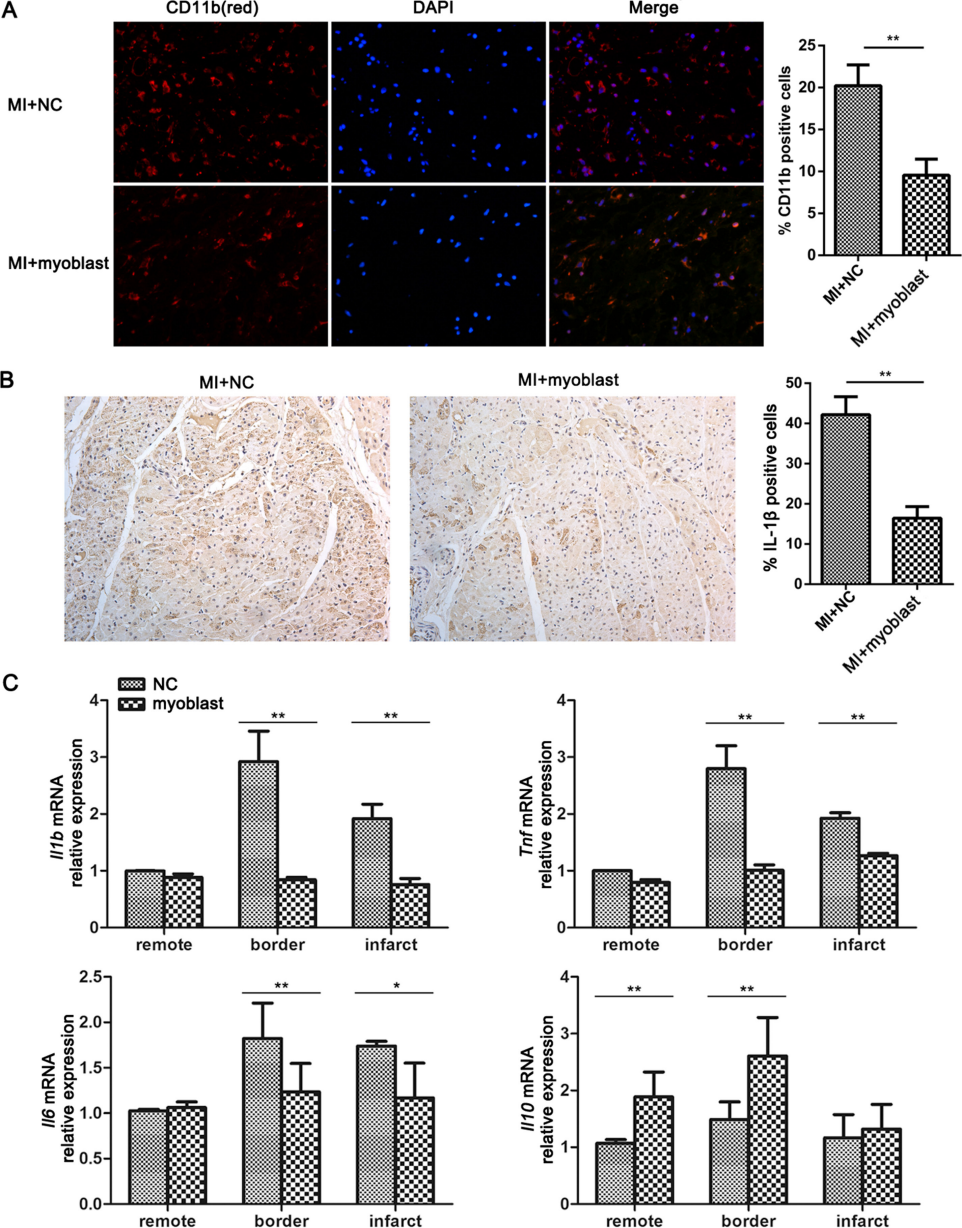
Myoblast transplantation attenuates inflammation in heart tissue post-MI (**A**) Immunofluorescence analysis of CD11b in heart sections from minipigs with MI followed by myoblast transplantation or control treatment (NC), and quantification of CD11b-positive cells is showed as a percentage of total cells counted. (**B**) Immunohistochemical staining of IL-1b in heart sections from minipigs treated as in (A), and quantification of IL-1b-positive cells is showed as a percentage of total cells counted. Original magnification, ×400. (**C**) Q-PCR analysis of the mRNA expression of IL-1β, TNF-α, IL-6 and IL-10 in different zones of heart tissues from minipigs treated as in (A). *n* = 6. Data are representative (A left, B left), or mean ± SEM (A right, B right, C) of 3 individual experiments. *P < 0.05, **P < 0.01.

Next we investigated the effect of myoblast transplantation on inflammatory responses in heart tissue post-MI. Immunofluorescence staining showed that the infiltration of CD11b positive inflammatory cells (monocytes/macrophages) was significantly decreased in heart tissue from myoblast transplantation group after MI compared with control treatment group (Figure 4A). Furthermore, immunohistochemistry staining showed that IL-1β production was also markedly suppressed in heart tissue of minipig post-MI with myoblast transplantation (Figure 4B). The expression levels of cytokines were further detected in remote, border and infarct zone of heart tissue with or without myoblast transplantation. As shown in Figure 4C, the mRNA expression levels of *Il1b*, *Tnf* and *Il6* were significantly down-regulated in border and infarct zone, while the expression of anti-inflammatory cytokine gene *Il10* was up-regulated in remote and border zone of heart tissue with myoblast transplantation, indicating that the grafted myoblasts can enhance the expression of anti-inflammatory cytokine IL-10 and in turn inhibit inflammatory cytokine production triggered by MI.

**Figure 5:**
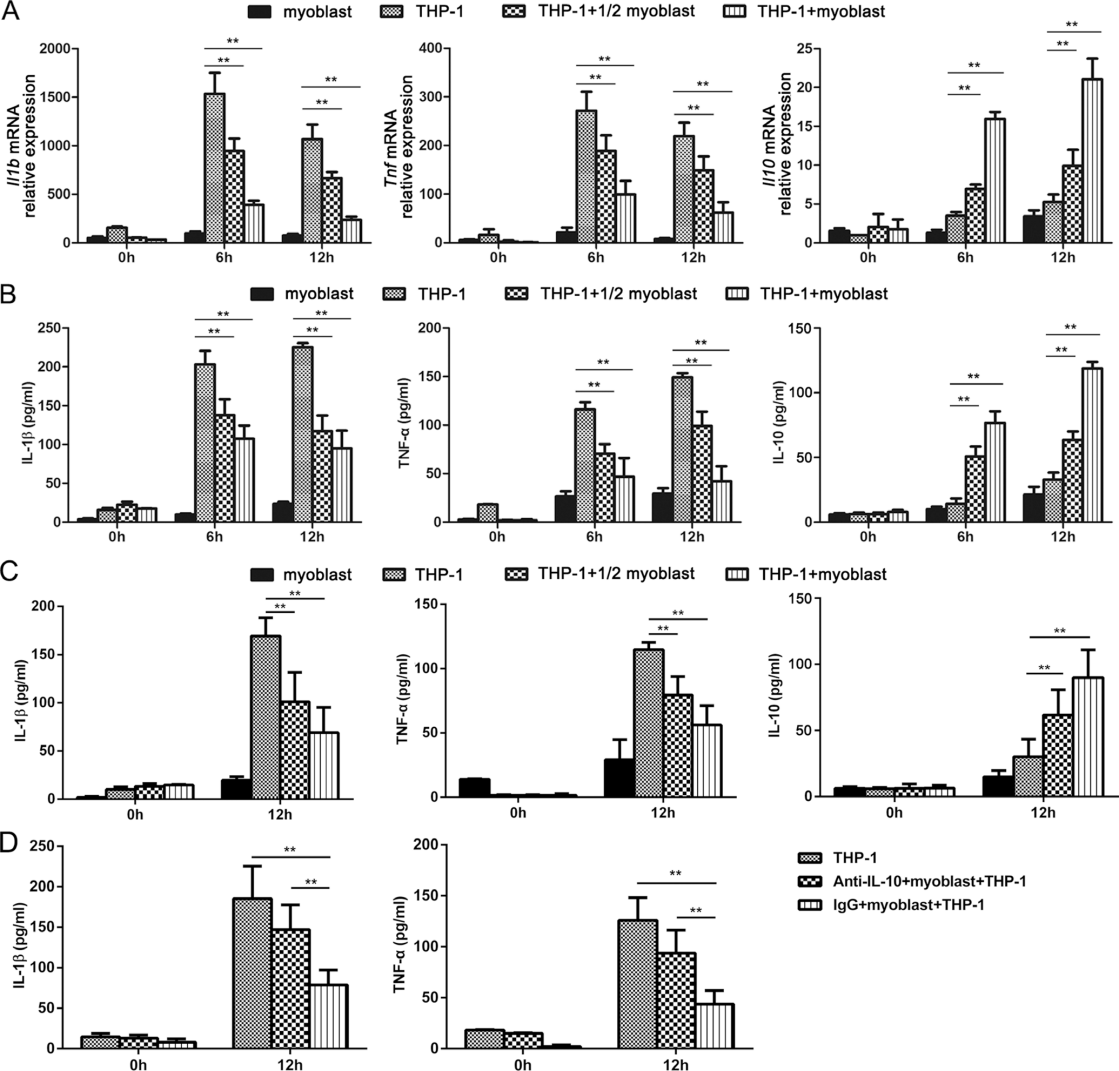
Myoblast co-culture decreases inflammatory cytokine production but increases IL-10 production triggered by DAMPs in THP-1 cells (**A**–**C**) THP-1 cells were cultured alone or co-cultured with the half or same number of myoblasts, and then stimulated with rhHMGB1 (A, B) or rhHSP60 (C) for the indicated times, the mRNA (A) and protein (B, C) expression levels of IL-1β, TNF-α and IL-10 were detected by Q-PCR analysis (A) and ELISA (B, C) respectively. (**D**) THP-1 cells were cultured alone or co-cultured with the same number of myoblasts, and then treated with IL-10 blocking antibody or IgG. The production of IL-1β and TNF-α triggered by rhHMGB1 in supernatant was detected by ELISA. Data are mean ± SEM of 3 individual experiments. **P < 0.01.

As we known, DAMPs including HMGB1 and HSP60 were released to trigger sterile inflammation after MI [24, 25]. The principle cells which recognize DAMPs and produce inflammatory cytokines post-MI are macrophages [26]. So we utilized a co-culture system with human myoblasts and human macrophage cell line THP-1 to explore the effect of myoblasts on DAMP-triggered production of inflammatory cytokines in macrophage. As shown in Figure 5A–5C, the mRNA and protein expression levels of IL-1β and TNF-α triggered by recombinational human HMGB1 (rhHMGB1) and recombinational HSP60 (rhHSP60) in THP-1 cells were significantly decreased when co-culturing with myoblasts in a cell number-dependent manner. However, co-culture with myoblasts had no obvious effect on IL-6 production triggered by rhHMGB1 and rhHSP60 in THP-1 cells (data not shown). In addition, myoblasts co-culture significantly promoted IL-10 production triggered by rhHMGB1 and rhHSP60 in THP-1 cells (Figure 5A–5C), which is consistent with the *in vivo* data from myoblast transplantation (Figure 3A and Figure 4C). What's more, we found that IL-10 blocking antibody treatment abolished the inhibitory effect of myoblast co-culture on the production of IL-1β and TNF-a triggered by rhHMGB1 in THP-1 cells (Figure 5D), confirming that the decreased production of inflammatory cytokines in macrophages co-cultured with myoblasts is attributed to the increased IL-10 production.

### Myoblasts inhibit the activation of MAPK and NF-kB pathway

**Figure 6:**
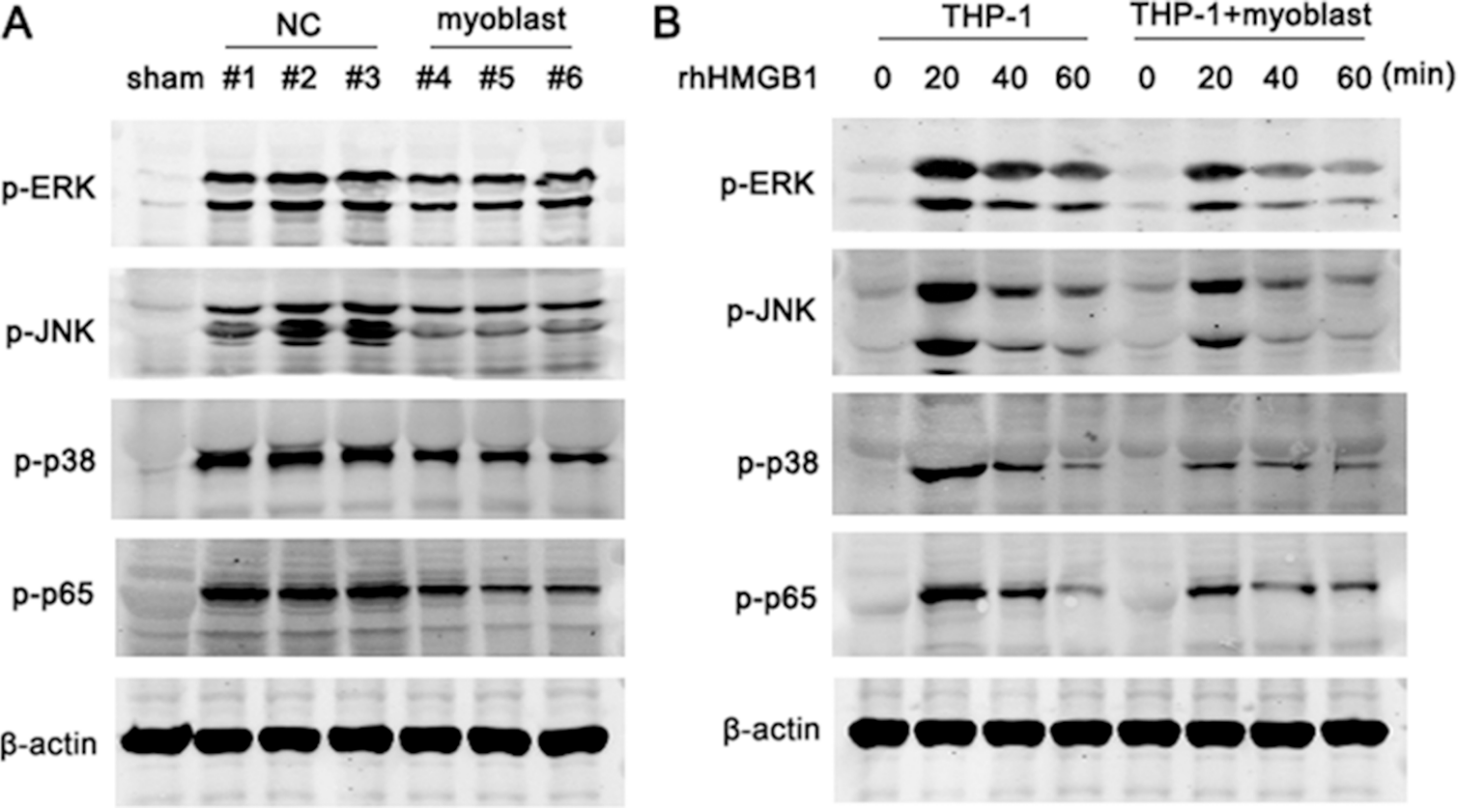
Myoblast inhibits the activation of MAPK and NF-kB pathway (**A**) Immunoblotting analysis of phosphorylation (p-) levels of ERK, JNK, p38 and p65 in lysates of heart tissues from minipigs with MI followed by myoblast transplantation or control treatment (NC). (**B**) Immunoblotting analysis of phosphorylation (p-) levels of ERK, JNK, p38, and p65 in lysates of THP-1 cells cultured alone or co-cultured with myoblasts followed by treatment with rHMGB1 for the indicated times. Data are representative of 3 individual experiments.

Previous studies have demonstrated that MAPK and NF-kB signaling pathway are required for sterile inflammation activation post-MI [26], we then investigated whether myoblast transplantation affected MAPK and NF-kB pathway activation. The phosphorylation levels of ERK, JNK, p38 and p65 were markedly decreased in heart tissue from minipig post-MI with myoblast transplantation compared with control treatment group (Figure 6A). Furthermore, co-culture with myoblasts also impaired the phosphorylation levels of ERK, JNK, p38 and p65 triggered by rhHMGB1 in THP-1 cells (Figure 6B). The results indicate that the impaired activation of MAPK and NF-κB signaling is contributed to the suppressed inflammatory response mediated by myoblast transplantation after MI.

## DISCUSSION

The in-depth study of the mechanism by which myoblasts improves cardiac function post-MI will benefit the application of myoblast transplantation. In this study, we found that the grafted myoblasts survived successfully in peri-infarct area and had important roles in promoting infarct healing and improving cardiac function. Microarray analysis indicated that many inflammation-related genes differentially expressed in peri-infarct heart tissues between groups of myoblast transplantation and control treatment, especially some pro-inflammatory cytokines down-regulated and anti-inflammatory cytokines up-regulated in myoblast transplantation group. Our study further demonstrates the novel function of myoblasts in the negative regulation of inflammatory response and uncovers the close relationship between inflammation and cardiac function after myoblast transplantation post-MI, which will has an instructional effect on the future clinical study.

Sterile inflammation induced by ischemia heart injury is a critical component of tissue healing. However, excessive activation of inflammation-related processes leads to adverse LV remodeling after MI. Some preclinical studies indicate that the expression levels of pro-inflammatory cytokines IL-6, TNF-α and IL-1β are closely associated with LV end-diastolic diameter measured at study termination [27]. One multicenter clinical trial of 1,200 patients with progressed heart failure shows that the up-regulated circulating levels of TNF, IL-6 and the soluble TNF receptors sTNFR1 and sTNFR2 are related with increased mortality [28]. Thus, inhibition of the myocardial inflammation is effective to attenuate cardiac dysfunction and prevent adverse cardiac remodeling after MI.

The injury incurred by cardiac myocytes and the extracellular matrix consequent to acute ischemic damage rapidly release DAMPs, which play a critical role in the sterile inflammatory response in MI by binding to pattern recognition receptors on or in cells of the innate immune system [29]. TLR4 is a key pattern recognition receptor, which can bind several DAMPs and initiates multiple inflammatory cascades [30]. In our microarray data, the expression of TLR4, as well as its downstream signal molecule MAPK8, significantly decreased in peri-infarct heart tissues with myoblast transplantation, which suggest that myoblasts limit recruitment of inflammatory cells to the injured myocardium and attenuate activation of inflammatory signal pathway. Forkhead box O1 (FOXO1), a transcription factor that functions in the regulation of cell proliferation, apoptosis and cardiovascular function. FOXO1 can promote inflammatory responses in innate immune cells triggered by TLR4 [31, 32]. Our microarray data found that FOXO1 expression was decreased in heart tissue with myoblast transplantation. These results suggest that FOXO1 may also be involved in the inhibition of inflammation mediated by grafted myoblasts. Thus, except for higher IL-10 production, the decreased expression of inflammation-related protein and suppressed activation of signaling pathway also attribute to the limited inflammation in heart tissue of minipig with myoblast transplantation.

Monocytes and macrophages are emerging as key players in mediating both the pathogen responses and sterile inflammation including that arising from ischemia heart injury [26, 33]. Cardiac macrophages are abundant in heart tissue after MI, which are mainly derived from circulating monocytes produced by the haematopoietic system and are centrally involved in inflammatory tissue remodeling, resolution of inflammation during post-MI healing, and left ventricular remodeling [26]. In our study, we demonstrate that myoblasts inhibit DAMP-triggered production of pro-inflammatory cytokines in macrophages through the increased IL-10 production *in vitro* co-culture system, together with the higher IL-10 gene expression in heart tissue with myoblast transplantation, our results suggest that myoblasts inhibit the inflammatory cytokine production in immune cells mainly including monocytes and macrophages in heart tissue through the higher IL-10 level and decreased activation of inflammation-related signaling pathway.

In summary, myoblasts have been shown as a most possible cell source for clinical applications such as ischemic cardiomyopathy through recent advances in research [34–36]. Our study further uncovers the novel mechanism underlying the improved cardiac function and prevention of adverse cardiac remodeling mediated by myoblast transplantation. The change of grafted cell number and implantation of myoblasts genetically engineered to over-express some factors will acquire more attractive and dramatic improvement in myoblast therapy.

## MATERIALS AND METHODS

### Cell culture

Human THP-1 cells were from the American Type Culture Collection and cultured in RPMI-1640 medium (Corning, Manassas, VA) supplemented with 10% fetal bovine serum (Gibco, Paisley, PA). Male human skeletal myoblasts were provided by the Cell Therapy Institute of Wuhan in China, and were cultured in Dulbecco's modified Eagle's medium (DMEM) (Corning) with 4.5 g/L of glucose. Cells were maintained in standard cell culture environment (95% humidity, 5% CO*2* at 37°C). Before transplantation, myoblasts were harvested and suspended in DMEM medium without phenol red and enriched in 2% BSA (Sigma-Aldrich, USA). Final concentration of myoblasts was 10^4^/μl of DMEM.

### Animal model and myoblast transplantation

Four-month-old Female Chinese Bama minipigs (16–20 kg) were from Taihe Biotechnology Co. (Taizhou, China). All minipigs were clinically healthy, housed in the animal facility under standard conditions and received humane care. All animal experiments were performed according to the National Institute of Health Guide for the Care and Use of Laboratory Animals, with the approval of the Scientific Investigation Board of Tongji University, Shanghai. Female minipigs were inductively anesthetized by intramuscular injection with midazolam (2 ml:2 mg) and ketamine (2 ml:0.1 mg), then endotracheal intubated, lastly connected to a ventilator (fre 20 TV 400ml I:E 1:1.5 FiO2 40%) assisted by propofol pump (20 ml:200 mg) for intra-operative maintain. Transthoracic small incision on left fourth intercostal space makes left circumflex (LCx) coronary artery visualized. There was no distinctive anatomic variation of LCx among the experiment minipigs. Preventative ligation (used a silk tie placed around the proximal LCx to reduce the outer diameter of the artery such that resting flow through the stenotic region was decreased by 80%, in this way tied 2 minutes and untied for 5 minutes) was performed three to five times, intravenous lidocaine was included in case of ventricular arrhythmia. Model of myocardial infarction was finally established by permanent ligation beyond the root of left circumflex coronary artery. After MI, 5 ml DMEM with or without 5×10^7^ myoblasts were intramyocardially injected into the promising infarct border zone with 10 separate injections using a 27-gauge needle. A total of 24 Bama minipigs were randomized into 4 groups (n = 6): Sham operation, MI group, DMEM injection after MI (control treatment), myoblast transplantation after MI. In addition, mice in control treatment group and myoblast transplantation group both received the same does of Cyclosporin A (5 mg/kg) before myoblast transplantation, whereas mice in sham group and MI group did not received Cyclosporin A. Electrocardiographic recordings revealed the short arrhythmia episodes occurred in a few minipigs of myoblast transplantation group within 7 days after myoblast transplantation or control treatment group, but these were self-limited and haemodynamically well tolerated.

### Histology

Triphenyl tetrazolium chloride (TTC) was used to determine the infarct size. Briefly, the whole heart was excised and quickly frozen for 20 minutes. The heart was cut into 5–10 mm transverse slices and then immersed in 1%-2% TTC solution for 30 minutes at 37°C. After fixed in 4% formalin, the slices were taken photo by digital camera and the infarct size was analyzed by Image J (National Institutes of Health, Bethesda, MD). HE staining was performed to assess the myocardial cell proliferation and inflammatory cell infiltration. Briefly, the heart was harvested and fixed by 4% paraformaldehyde, embedded in paraffin. Serial sections were cut into 6 μm thickness and stained with hematoxylin & eosin and then observed with Leica microscope. The heart sections were also subjected to immunohistochemistry staining with IL-1β antibody (ebioscience).

### Immunofluorescent analysis

Minipig heart was excised and fixed by 4% paraformaldehyde. Serial sections were cut into 6 μm thickness to prepare for immunofluorescence analysis. Sections were incubated with primary anti-human myosin heavy chain antibody (Millipore), anti-human histocompatibility antigen class I antibody (Abcam) and anti-minipig CD11b antibody (ebioscience) followed by incubation with Alexa Fluor-conjugated secondary antibody (Thermo Scientific). DAPI was used to label total cells. Cells were examined under a confocal laser microscope (Leica TCS SP5II STED, Mannheim, Germany).

### Two-dimensional speckle tracking echocardiography analysis

Transthoracic Echocardiography was performed in the anesthetized minipigs using Vevo2100 (Visual Sonics VSI, Toronto, ON, Canada) with MS400 linear array transducer (38 MHz). Cardiac function parameters were obtained from short-axis and long-axis views of speckle tracking echocardiography.

### Microarray analysis

Total RNA was extracted using TRIZOL Reagent (Invitrogen, Carlsbad, CA) and checked for a RIN number to inspect RNA integrity by an Agilent Bioanalyzer 2100 (Agilent technologies, Santa Clara, CA). Qualified total RNA was further purified by RNeasy micro kit (QIAGEN, Hilden, Germany) and RNase-Free DNase Set (QIAGEN). Biotin-labeled cRNA was generated with the GeneChip 3′IVT Express Kit (Affymetrix, Santa Clara, CA) for the Affymetrix system, and then hybridized to GeneChip^®^ Porcine Genome Arrays (Affymetrix), containing 23,937 probe sets to examine 23,256 transcripts, and scanned by GeneChip^®^ Scanner 3000 (Affymetrix, Santa Clara, CA) and Command Console Software 3.1 (Affymetrix, Santa Clara, CA). Raw data were normalized by MAS 5.0 algorithm, Gene Spring Software 11.0 (Agilent tech, Santa Clara, CA). The genes with significant differential expression (a fold change > 2) between the 2 different groups were filtered.

GO analysis and KEGG analysis were applied to determine the biological roles of these differentially expressed mRNAs, based on the latest KEGG (Kyoto Encyclopedia of Genes and Genomes) database (http://www.genome.jp/kegg/). Inflammation-related Genes annotated by GO terms (http://www.geneontology.org/) and KEGG pathway were filtrated out and visualized as a heatmap chart and scatter plot based on expression level. Array data are available at Gene Expression Omnibus (http://www.ncbi.nlm.nih.gov/geo/) under the accession number GSE 94151.

### RNA isolation and quantitative PCR

Total RNA was isolated from heart tissues and cells using Trizol reagent (Invitrogen, Carlsbad, CA). cDNA was synthesized by reverse transcription synthesis kit (TOYOBO, Osaka, JAPAN). The expression of human Y chromosome was detected with the change of sex determining region Y (SRY). The gene expression levels of *Tnf*, *Il1b*, *Il6* and *Il10* were analyzed by quantitative PCR (Q-PCR) using SYBR Green PCR kit (TOYOBO, Osaka, Japan) and ABI 7900 (Applied Biosystems, Waltham, MA). The primers for the tested genes were purchased from JIELI Biology (Shanghai, China) and their sequences were showed in Table 3.

**Table 3:** Q-PCR primers for detection of mRNA expression of cytokines

Gene symbol	Primers
Human *IL1b*	F: AGCTACGAATCTCCGACCAC R: CGTTATCCCATGTGTCGAAGAA
Human *IL10*	F: GACTTTAAGGGTTACCTGGGTTG R: TCACATGCGCCTTGATGTCTG
Human *TNF*	F: ATGAGCACTGAAAGCATGATCC R: GAGGGCTGATTAGAGAGAGGTC
Human *Sry*	F:GCGTATTCAACAGCGATGATTAC R:TCTCCCGTTTCACACTGATACTT
Minipig *Il1b*	F: AGGTCCACATGGGCTGAAGAAC R: GGCTGGCTTTGAGTGAGGAGAA
Minipig *Il10*	F: CTGAGAACAGCTGCATCCAC R: TGGCTTTGTAGACACCCCTC
Minipig *Tnf*	F: GCTGTACCTCATCTACTCCC R: TAGACCTGCCCAGATTCAGC

### Enzyme-linked immunosorbent assay (ELISA)

Cytokine (IL-1β, IL-6, IL-10, TNF-α) production in supernatants of cell culture were assayed using ELISA kit (R&D, Minneapolis, MN) according to manufacturer's instructions.

### Immunoblotting

Heart tissues and cells were lysed with cell lysis buffer (Cell Signaling Technology) containing protease inhibitor mixture (Merck Millipore). Protein concentration of the extracts was measured with BCA assay (Thermo Fisher Scientific). Immunoblotting analysis was performed as described previously [37].

### Statistical analysis

Data were presented as mean ± standard error of the mean (SEM). The unpaired Student's *t*-test and one-way ANOVA were used to assess the significance of differences between data. *P* < 0.05 was considered to be statistically significant.

## SUPPLEMENTARY MATERIALS FIGURE


