# Supplementary material for: Myoblast transplantation improves cardiac function after myocardial infarction through attenuating inflammatory responses
Source: Oncotarget. 2017 May 27;8(40):68780–94. doi: 10.18632/oncotarget.18244 (PMC5620296; doi:10.18632/oncotarget.18244)
Supplement: Supplementary file 1 [file oncotarget-08-68780-s001.pdf]

Myoblast transplantation improves cardiac function after myocardial infarction through attenuating inflammatory responses

Supplementary Materials

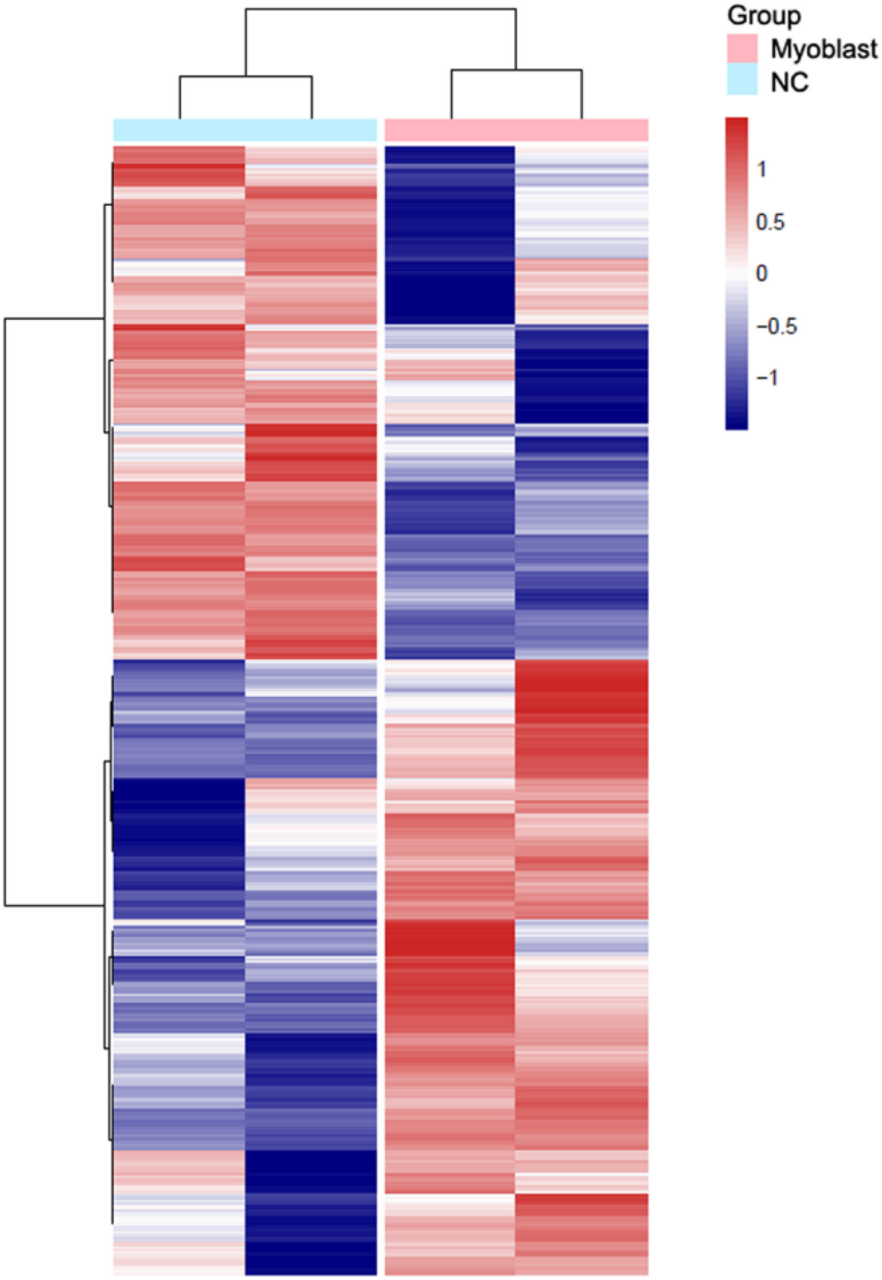

Supplementary Figure 1: Heat map showing hierarchical clustering of differentially expressed transcripts in heart tissues from minipigs with MI followed by myoblast transplantation or control treatment (NC).
